# The protein kinase R modifies gut physiology to limit colitis

**DOI:** 10.3389/fimmu.2023.1106737

**Published:** 2023-02-17

**Authors:** Howard Chi Ho Yim, Arindam Chakrabarti, Sean Kessler, Hiroyuki Morimoto, Die Wang, Dhanya Sooraj, Afsar U. Ahmed, Carol de la Motte, Robert H. Silverman, Bryan RG. Williams, Anthony J. Sadler

**Affiliations:** ^1^ Centre for Cancer Research, Hudson Institute of Medical Research, Clayton, VIC, Australia; ^2^ Department of Molecular and Translational Science, Monash University, Clayton, VIC, Australia; ^3^ Department of Cancer Biology, Lerner Research Institute, Cleveland, OH, United States; ^4^ Department of Pathobiology, Lerner Research Institute, Cleveland, OH, United States; ^5^ Department of Anatomy, School of Medicine, the University of Occupational and Environmental Health, Kitakyushu, Fukuoka, Japan; ^6^ Centre for Innate Immunity and Infectious Diseases, Hudson Institute of Medical Research, Clayton, VIC, Australia

**Keywords:** colitis, protein kinase, inflammasomes, autophagy, goblet cells, inflammation, gut barrier

## Abstract

Here we investigate the function of the innate immune molecule protein kinase R (PKR) in intestinal inflammation. To model a colitogenic role of PKR, we determine the physiological response to dextran sulfate sodium (DSS) of wild-type and two transgenic mice strains mutated to express either a kinase-dead PKR or to ablate expression of the kinase. These experiments recognize kinase-dependent and -independent protection from DSS-induced weight loss and inflammation, against a kinase-dependent increase in the susceptibility to DSS-induced injury. We propose these effects arise through PKR-dependent alteration of gut physiology, evidenced as altered goblet cell function and changes to the gut microbiota at homeostasis that suppresses inflammasome activity by controlling autophagy. These findings establish that PKR functions as both a protein kinase and a signaling molecule in instituting immune homeostasis in the gut.

## Introduction

Inflammatory bowel disease (IBD) is a heterogeneous disorder that is commonly characterized as either ileal or colonic Crohn’s disease, or ulcerative colitis. The precise mechanisms of how disease manifests remain to be established, but IBD is considered to be a consequence of the loss of immune tolerance against the gut microbiota. Current anti-inflammatory and immunosuppressive treatments provide only temporary relief and are not universally effective. Greater mechanistic insights into gut immunity is required in order to develop strategies to control immune pathogenesis.

The protein kinase R (PKR) is a member of the small family of eukaryotic initiation factor alpha (eIF2α) kinases that constitute a universal stress response in eukaryotes (1). Among this kinase family PKR is most cogently linked with immunity, as its expression is induced by the antiviral type I and III interferons. These cytokines, particularly the type III interferons, are important for epithelial function and mucosal immunity (2–5). The function of PKR in colitis is currently confused, with discordant effects reported (6, 7). This warrants further study.

Here we reassess the role of PKR in dextran sulfate sodium (DSS)-induced colitis using mice that are ablated for PKR expression. We replicate experiments conducted in previous studies but investigate an alternative mode of activity from that previously proposed, identifying a different mechanism of PKR activity in colitis. Rather than PKR functioning through induction of the unfolded protein response (UPR), which is more closely associated with the related PKR-like endoplasmic reticulum kinase (PERK), we contend that PKR promotes gut barrier function and suppresses inflammatory pathogenesis in colitis by controlling autophagy in goblet cells. Additionally, we test the response of a transgenic mouse with a point mutation that disables the kinase activity of PKR, thereby testing functions that are independent of the kinase’s canonical control of the initiation of translation. The findings demonstrate that PKR functions in DSS-induced pathogenesis by suppressing the activity of inflammasomes through modulation of the gut physiology by kinase-dependent and -independent processes. This accords with the reported activity of another eIF2α kinase, the general control nonderepressible 2 (GCN2) in DSS-induced colitis (8), although this response had not been segregated from its phosphorylation of eIF2α. These findings reinforce the importance of autophagy to promote gut barrier function in gastric disease, particularly by supporting the function of goblet cells.

## Materials and methods

### Mice

Congenic C57BL/6J mice (8-10 weeks old) were exposed to 2.5% DSS (36-50 kDa, MP Biomedicals) in their drinking water for up to 9 days, with or without intraperitoneal injection of 2 mg/kg of CP456773 (Sigma-Aldrich) prior to DSS treatment. Separately reared wild-type (WT), PKR-ablated (*Eif2ak2* ^-/-^) and kinase-dead PKR (K271R) mice were treated with DSS at the Hudson Institute of Medical Research in Australia. This experiment was extended with a second cohort of littermate WT and *Eif2ak2* ^-/-^ mice reared at the Learner Research Institute in the USA. Experiments were performed according to the Guide for the Care and Use of Laboratory Animals of the National Institutes of Health following a protocol that was approved by the Institutional Animal Care and Use Committee of Cleveland Clinic, or the Australian Code of Practice following a protocol approved by the Monash Medical Centre Animal Ethics Committee. *Eif2ak2* ^-/-^ and K271R mice were produced as previously reported (9, 10). A disease activity index (DAI) was calculated as described previously (11).

### Histology

Tissues from the stomach, small intestine and colon were cut open, rinsed with PBS, then fixed in 10% formalin for 4 hours before embedding in paraffin. Embedded tissue was sectioned (5 μm) then stained with: biotinylated hyaluronan-binding protein (Calbiochem-EMD Millipore) and streptavidin-488 (Life Technologies), anti-alpha actin 2 and alexa fluor 568 then mounted with Vectashield containing DAPI as previously described (11); hematoxylin and eosin (H&E); anti-proliferating cell nuclear antigen (PCNA), -villin and -H^+^/K^–^ATPase primary antibodies and alexa fluor 546 or 488 donkey anti-goat or -mouse secondary antibodies (Molecular Probes); or alcian blue/periodic acid–Schiff (PAS). Stains were detected with TSA Fluorescence System (Perkin Elmer), imaged by Axioskop 2 plus fluorescence microscope (Carl Zeiss) and analyzed with AxioVIson SE64 software (Carl Zeiss) or ScanScope XT digital scanner (Leica) and ImageScope software (Leica) and quantified from four random images per mouse using ImageJ software (NIH). Sections were scored blindly. Colitis was scored as previously described (11).

### *In situ* hybridization

*In situ* hybridization was performed with the Falma microprobe system as stipulated by the manufacturer (Falma). Sections were hybridized with digoxigenin (DIG)-labeled control sense and anti-sense RNA probes encompassing nucleotides 173 to 1091 of the open-reading frame of the PKR gene (*Eif2ak2*). Sections were immunohistochemically stained to visualize the hybridized probe using AP-conjugated mouse monoclonal anti-DIG antibody (Roche) and either the Fast Red or NBT/BCIP substrates for stomach or colon tissues, respectively (ThermoFisher Scientific). Images were captured by fluorescence microscopy.

### Flow cytometry

Spleens were mashed in RPMI 1640 (SIGMA) containing 1% fetal bovine serum (FBS), strained through a nylon filter, then washed before staining with FAM-FLICA according to the manufacturer’s instructions (ImmunoChemistry Technologies LLC), washed with PBS then stained with anti-F4/80 Pacific Blue antibody (MCA497PB Bio-Rad) and anti-Ly6G APC-Cy7 antibody (BD Biosciences), washed with PBS and fixed in 10% formalin then visualized by BD FACSCanto II and analyzed by Cytobank software (Cytobank Inc.)

### Colon explant

Tissue pieces (0.5 cm) were cut from the proximal colon and rinsed with PBS, then cultured in DMEM containing 1% FBS and penicillin/streptomycin at 37°C for 24 hours. The level of tumor necrosis factor α (TNFα) was assayed by ELISA (555268 BD Biosciences). The cell-free supernatant (500 μl) was precipitated by adding methanol and chloroform as previously reported (12), then probed with anti-interleukin (IL)1β (ab9722 Abcam) and -IL18 (D046-3 MBL International) antibodies by immunoblot.

### Immune fluorescence

Caspase-1 (Casp1) activity was assessed by FAM-FLICA and FLICA-660 (No. 97 and 9122, respectively, from ImmunoChemistry Technologies LLC or, alternatively, ThermoFisher Scientific). The fluorescent reporter was quantitated in immune cells isolated from the spleen by flow cytometry or, alternatively, in tissues by confocal microscopy after cryopreservation, sectioning (5 μm), being fixed with 10% formalin and permeabilized by methanol. The mucus layer in the colon and goblet cells was visualized with a fluorescein-linked lectin *Ulex europaeus* agglutinin-1 (UEA1) (ThermoFisher Scientific). Mucus thickness was measured in micrographs by confocal microscopy. Autophagic puncta were detected in fixed and permeabilized colon tissues with a fluorescent antibody to the microtubule-associated protein 1A/1B light chain 3B and (LC3B) (ThermoFischer Scientific). Autophagosomes in goblet cells were scored as LCB and UEA1 positive cells. Cells and tissues were counterstained with Hoechst 33342 (Merck) to visualize cell nuclei. Images were captured by Nikon C1 confocal microscope and analyzed by Imaris software.

### Immunoblot

Protein lysates from splenic cells were harvested by RIPA buffer as previously described (10), heat denatured in sample buffer (125 mM Tris-HCl, pH 6.8, 4% SDS, 20% glycerol, 5% β-mercaptoethanol, 0.01% bromophenol blue) and resolved through 10-15% SDS-polyacrylamide gel by electrophoresis, then transferred to Immobilon-FL membrane (Millipore). Membranes were treated with blocking buffer (LI-COR) then probed with primary and secondary antibodies conjugated with fluorophore, and visualized and quantified using the Odyssey Imaging System (LI-COR). Lysates were probed with anti-IL1β, -IL18, -CASP1-p10 (sc-514 Santa Cruz Biotechnology Inc.), anti-apoptosis-associated speck-like protein containing a CARD (ASC) and -NOD-, LRR- and pyrin domain-containing protein 3 (NLRP3) antibodies (AL177 and Cryo-2, respectively, *Adipogen*), -eukaryotic initiation factor 2 (Eif2) and -phospho-Eif2 Ser51) (#9722 and 119A11, respectively, Cell Signaling Technology) *and* protein loading was assessed with anti-β-actin (ab8226 Abcam) or -α-tubulin (3873 Cell Signaling) antibodies.

### Affinity chromatography

To enrich the kinase domain of PKR, protein lysates of murine fibroblasts were captured on a Hi-Trap heparin column following the manufacturer’s protocol (GE Life Sciences). Eluted peptides were separated by PAGE and transferred to a membrane support before being probed with the anti-PKR antibody D-20 (Santa Cruz Biotechnology).

### Bacterial content detection in feces

DNA was extracted by the QIAmp Fast DNA Stool Mini Kit, following the manufacturer’s protocol (QIAGEN) and 1 pg was used with previously described primers (13) to amplify the 16s rRNA gene of *Bacteroides, Lactobacillus* and *Prevotella* by quantitative PCR using Applied Biosystems 7900HT systems.

### Statistical analysis

The Prism software (GraphPad) was used for all statistical analyses. Statistical significance of the differences between two groups was analyzed by two-way or one-way ANOVA with either with Šidàk post-test or Tukey’s range test or, alternatively, two-tailed and unpaired Student’s t-test. The correlation analysis was done by one-tailed Pearson correlation test.

## Results

### PKR affects DSS-induced weight loss

**Figure 1:**
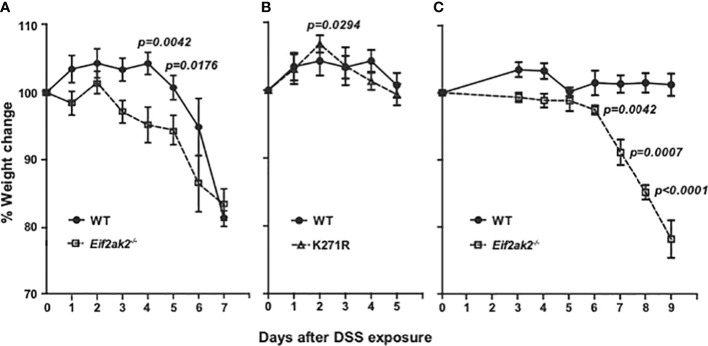
PKR reduces DSS-induced weight loss. **(A–C)** Body weight of mice treated with 2.5% DSS in their drinking water, expressed as the percentage change from the starting weight of: **(A)** Separately reared WT and PKR-ablated (*Eif2ak2^-/-^*) mice (n=19 and 13, respectively); **(B)** WT compared to PKR kinase-dead (K271R) mice (n=19 and 7, respectively), and; **(C)** Cohoused WT and *Eif2ak2* ^-/-^ littermates (n=5 and 3, respectively). Data were collected from three independent experiments and are expressed as the mean ± S.E.M. and analyzed by two-way ANOVA with Šidàk post-test on the means between genotypes on each day.

Cohorts of congenic WT and PKR mutant mice were treated with DSS in their drinking water and their respective weights compared. The *Eif2ak2* ^-/-^ mice show significantly greater weight loss compared to the WT animals after the second day of DSS treatment until day 6 (**Figure 1A**). This increased weight loss in the first five days of exposure to DSS was not apparent in the kinase-dead K271R mice (**Figure 1B**). Accordingly, PKR expression, independent of substrate phosphorylation, is protective against DSS-induced weight loss.

These experiments were conducted on mice reared in separate cages at the Monash Animal Research Platform at Monash University, Victoria, Australia. The response to DSS is strongly influenced by environmental variables, particularly differences in the microbiota. This has been proposed as a cause of discrepancies between separate studies that use this model of colitis. To explore this contingency, we performed a second comparison with a limited number of WT and *Eif2ak2* ^-/-^ littermate mice raised at the Animal Core, Lerner Research Institute, Ohio, USA. These data confirm the protective function of PKR against this insult, although there were some changes (**Figure 1C**). Most conspicuously, the WT mice from the Lerner maintained their weight throughout the experiment. Additionally, the kinetics of weight loss in the PKR-ablated mice was delayed compared to the cohort from the Monash (**Figures 1A, C**).

These data identify that PKR protects against DSS-induced weight loss and distinguish kinase-dependent and -independent effects of PKR, as has been previously asserted from *in vitro* experiments (14).

### PKR affects DSS-induced tissue injury

**Figure 2:**
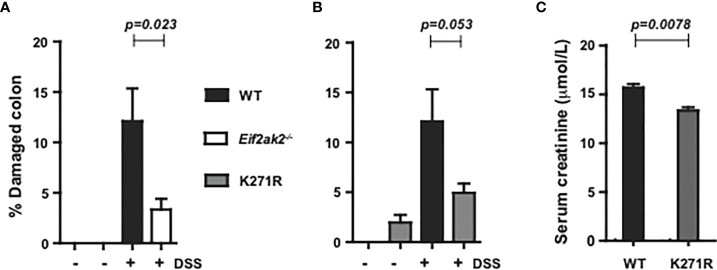
PKR kinase activity modulates DSS-induced tissue damage. The severity of colon damage of the mice reared separately at the Monash Animal Research Platform, expressed as the percentage of the entire length of the colon in either **(A)** WT compared to PKR-ablated (*Eif2ak2* ^-/-^) mice or **(B)** WT compared to the kinase-dead PKR mice (n=6). Data collected from two independent experiments are expressed as mean ± S.E.M. and analyzed by two-way ANOVA with Šidàk post-test on the mean between genotypes on each day. **(C)** Measures of the levels of creatinine in the serum of the indicated mice (n=3). Data are expressed as mean ± S.D. and analyzed by unpaired t-test from a single experiment.p=0.023; p=0.053; p=0.0078.

Only modest tissue pathogenesis was evident in tissue sections of the colon from any of the three mouse genotypes reared at the Monash Animal Research Platform. Accordingly, the entire length of the colon was assessed for damage by quantifying disorganized and incomplete crypts as a percentage of the longitudinal length of the colon (as previously described (15)). Tissues were assessed on day five of DSS treatment, when the differential in weight was greatest between the separate cohorts (**Figure 1A**). The WT animals showed increased tissue injury compared to both the PKR-ablated and kinase-dead (K271R) mice, although the difference between kinase-dead and WT mice was not assessed as being significantly different (**Figures 2A, B**). A quantitation of serum creatinine levels (measured at the Monash Health Pathology), which is used as a clinical variable of colonic injury, appears to confirm a worsened response in the WT compared to the kinase-dead mice (**Figure 2C**). These data identify a discordance between DSS-induced weight change (**Figure 1**) and tissue damage in the colon, which was mediated by PKR’s kinase activity (**Figures 2A, B**).

The equivalent analysis was not conducted on the cohort from the Lerner Research Institute, although an analysis of disease index with a histological analysis of tissues from the colon of the mice exposed to DSS for 9 days is shown as supplementary data (**Supplementary Figure 1**).

### PKR limits DSS-induced inflammation in the gut

**Figure 3:**
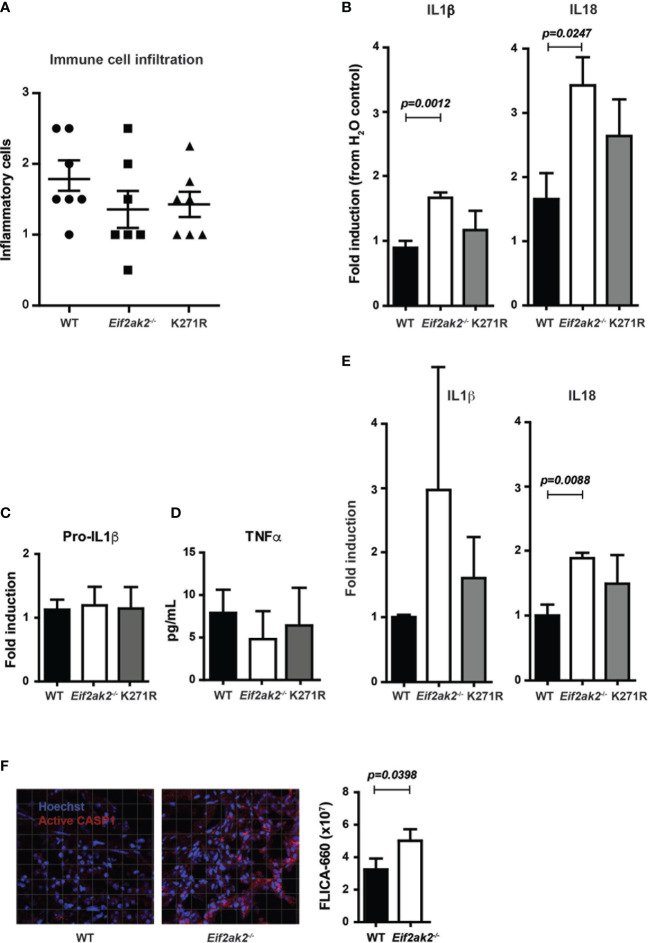
PKR limits DSS-induced inflammasome activity in the colon. **(A-F)** Analysis of inflammasome activity in WT, PKR-ablated (*Eif2ak2* ^-/-^) and kinase-dead (K271R) mice. **(A)** Inflammatory cell infiltrates into the colons of mice treated with DSS for 5 days assessed from H&E-stained tissue sections (n=7). Data were collected from two independent experiments. **(B)** Quantitation of the relative levels of cleaved IL1β and IL18 produced from colon explants of DSS-treated mice as detected by immunoblot (n=7). Data were collected from two independent experiments. **(C)** Induction of pro-IL1β in colon explants measured by immunoblot and expressed relative to WT mice (n=4). Data were collected from four independent experiments and analyzed by one-way ANOVA with Tukey’s range test. **(D)** Levels of TNFα expressed from colon explant detected by ELISA (n=4). Data were collected from four independent experiments and analyzed by one-way ANOVA with Tukey’s range test. **(E)** Quantitation of the relative levels of cleaved IL1β and IL18 produced from colon explants of untreated mice as detected by immunoblot (n=7). Data were collected from two independent experiments. **(F)** Micrographs of colon tissue from untreated mice stained for Casp1 activity (FLICA-660) (red) and counter stained with Hoechst to mark cell nuclei (blue). Casp1 activity is quantitated by fluorescent confocal microscopy in the graph on the right (n=3). Data is expressed as mean ± S.E.M. and analyzed by unpaired t-test.

We assessed the immune response in the DSS-treated cohort that was reared at the Monash Animal Research Platform. There was a modest, statistically nonsignificant, increase in the immune cell infiltrate into colon tissues from WT mice compared to the PKR-ablated or kinase-dead (K271R) mice (**Figure 3A**). However, there were significantly lower levels of the inflammatory IL1β and IL18 cytokines in the WT compared to the PKR-ablated mice (**Figure 3B**). The PKR-K271R mice had a lower, but not statistical different, level of these cytokines from that in the WT mice (**Figure 3B**). As the modestly heightened tissue damage in the WT mice is not accompanied by a commensurate increase in inflammatory markers, this damage would appear to be a consequence of primary injury by DSS, rather than being immune mediated. The data also identify that inflammasome activity is suppressed, partly but not entirely, by substrate phosphorylation by PKR. Notably, the expression of the inflammasome constituents; caspase-1 (Casp1), apoptosis-associated speck-like protein containing a CARD (Asc), and the NOD-, LRR- and pyrin domain-containing protein 3 (Nlrp3) was equivalent between the different mice (**Supplementary Figure 2**). There was also no difference in expression of the unprocessed pro-IL1β cytokine or the induction of the independent (of inflammasomes) inflammatory cytokine TNFα (**Figures 3C, D**). Assessment of the levels of cleaved cytokines in untreated mice indicated an increase in inflammasome activity in the PKR-ablated mice at homeostasis (**Figure 3E**). Correspondingly, a fluorescent substrate reporter indicated that there was heightened Casp1 activity in the colon tissue from PKR-ablated mice compared to the WT animals prior to treatment with DSS (**Figure 3F**).

These data identify that PKR represses inflammasome activity at homeostasis and limits cytokine processing in the colon in response to DSS treatment. The retention of much of this activity in the kinase-dead mouse identifies partial independence from eIF2α phosphorylation.

### PKR limits DSS-induced inflammation in neutrophils

**Figure 4:**
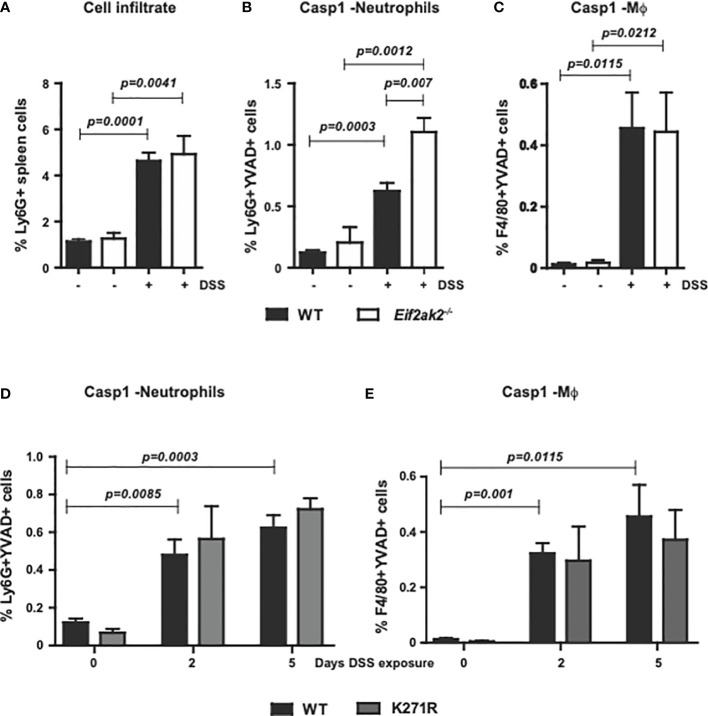
PKR limits inflammasome activity in splenic neutrophils. **(A)** Detection of Ly6G^+^ inflammatory cells in the spleens of WT compared to PKR-ablated (*Eif2ak2* ^-/-^) mice either untreated or treated with DSS for five days. **(B–E)** Measures of Casp1 activity (YVAD+), quantified by flow cytometry in splenic neutrophils (Ly6G^+^+YVAD^+^) and macrophages (Mϕ, F4/80^+^+YVAD^+^) from **(B, C)** the WT compared to PKR-ablated mice after 5 days of DSS treatment (H_2_O, n=4 and DSS, n=5) and **(D, E)** WT compared to PKR-K271R kinase-dead mice assessed after the indicated day of DSS treatment (n=6). Data are expressed as mean ± S.E.M. and analyzed by two-way ANOVA with Šidàk post-test.

To measure the immune response outside of the gut, we assessed innate immune cells from the spleens of WT, PKR-ablated and the kinase-dead mice treated with DSS for 5 days. Equivalent levels of the leukocyte antigen lymphocyte antigen 6 complex locus G6D (Ly6G) was apparent in the spleens of WT and *Eif2ak2* ^-/-^ mice at rest and this was uniformly elevated after treatment with DSS (**Figure 4A**). Comparison of the cleavage of a Casp1 substrate reporter (YVAD) in Ly6G-positive and adhesion G protein-coupled receptor E1 (F4/80)-positive cells from the spleens of mice showed that DSS treatment induced Casp1 activity in neutrophils disproportionately more in the PKR-ablated relative to the WT mice (**Figures 4B, C**). This PKR-dependent suppression of Casp1 activity was independent of eIF2α phosphorylation, as splenic neutrophils from the WT and kinase-dead mice demonstrated equivalent reporter activity (**Figures 4D, E**). Accordingly, the heightened inflammatory response caused by ablating PKR expression extends beyond the gastrointestinal tract.

### Multiple inflammasomes are active in DSS-induced inflammation

**Figure 5:**
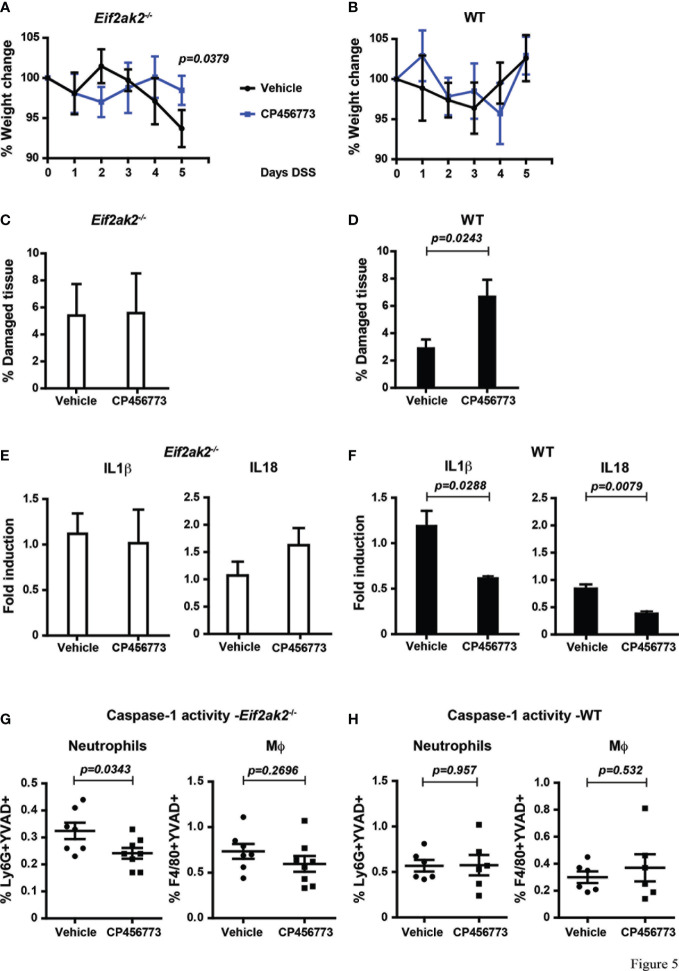
Inhibiting NLRP3 restores control in the absence of PKR expression. **(A, B)** Body weights of mice treated with DSS and injected with either the NLRP3 inhibitor CP456773 or the control vehicle solute, expressed as the percentage change from the starting weight of **(A)** PKR-ablated (*Eif2ak2* ^-/-^) or **(B)** WT mice (n=6) and analyzed by two-way ANOVA with Šidàk post-test. **(C, D)** Measures of the effect of CP456773 on DSS-induced tissue damage, expressed as the percentage of the entire length of the colon in either **(C)** PKR-ablated or **(D)** WT mice (n = 6) and analyzed by unpaired t-test. **(E, F)** Measures of the effect of CP456773 on the relative change in the levels of mature IL1β and IL18 produced from colon explants from either **(E)** PKR-ablated or **(F)** WT mice treated with the inhibitor (WT+Vehicle and WT+CP456773 n=3; *Eif2ak2* ^-/-^+Vehicle n=4; *Eif2ak2* ^-/-^+CP456773 n=5). Cytokines were assayed by immune blot and expressed as fold induction compared to Vehicle-treated mice and analyzed by unpaired t-test. **(G, H)** Casp1 activity (YVAD+) in splenic neutrophils (FLICA^+^Ly6G^+^) or macrophages (Mϕ, FLICA^+^F4/80^+^) from either **(G)** PKR-ablated or **(H)** WT mice treated with the NLRP3 inhibitor (n=7 and n=8, respectively). Fluorescent probes for Casp1 activity were detected and quantitated by flow cytometry. Data are expressed as mean ± S.E.M. and analyzed by unpaired t-test.

As PKR is controlling the activity of the inflammasomes and because NLRP3 has been shown to be important in colitis, we treated mice with the inhibitor CP456773 to assess the involvement of this sensor in the response to DSS (16). WT and PKR-ablated mice were injected with CP456773 or, as a control, the carrier solvent at days 1, 2 and 4 during the course of 5 days of DSS treatment. This treatment diminished the weight loss of the PKR-ablated mice, thereby confirming a function for NLRP3 in this phenotype (**Figures 5A, B**). Intriguingly, treatment with CP456773 worsened the low level of DSS-induced tissue damage in the WT animal but not in the PKR-ablated mouse (**Figures 5C, D**). This appears consistent with PKR kinase activity promoting tissue damage *via* suppression of the NLRP3 inflammasomes (**Figures 2**, **3**).

The NLRP3 inhibitor further reduced the low levels of IL18 and IL1β in colon explants from the WT animals but had no significant effect on the relatively higher levels of these cytokines in the PKR-ablated mice (**Figures 5E, F**). Accordingly, the processing of these cytokines in the colon from *Eif2ak2* ^-/-^ mice is mediated by an inflammasome constituted by a sensor protein other than NLRP3 (**Figures 5E** with **3B**). This finding with the data showing CP456773 stabilized the weight of mice treated with DSS (**Figures 5A, B**) also suggest that this alternative inflammasome is not causal of the observed weight loss (**Figures 5A, B** with **Figure 1**). Opposing this pattern in the colon, CP456773 reduced measures of Casp1 activity in splenic neutrophils from PKR-ablated mice, while there was no change in the WT mice (**Figure 5G, H**). Therefore, NLRP3 contributes to the inflammasome that is active in splenic neutrophils from *Eif2ak2* ^-/-^ mice.

These data identify PKR-dependent suppression of inflammasome activity and show that different sensor proteins constitute inflammasomes in separate tissues of the DSS-treated mice. Better maintenance of weight in CP456773-treated mice suggests that NLRP3 participates in this phenotype, despite no evidence of suppression of cytokines processed by inflammasome activity in the colon. In addition, the modest increase in tissue damage observed in the colon of CP456773-treated mice suggests that NLRP3 is protective against DSS-induced damage. A possible cause for this apparent altered susceptibility was investigated.

### PKR affects gastrointestinal physiology

We examined the gut physiology of WT, PKR-ablated and point mutant kinase-dead mice. Although at odds with the original description and subsequent measures (7, 9), it was reported that the mutated *Eif2ak2* locus retained expression of a truncated kinase domain (17). Accordingly, we sought to verify the ablation of PKR in the *Eif2ak2* ^-/-^ mouse. Whole cell lysates from embryonic fibroblasts from the WT and *Eif2ak2* ^-/-^ mice were passed through a heparin column to capture the putative peptide *via* its reported binding of heparin (18). The removal of PKR from the *Eif2ak2* ^-/-^ mouse was confirmed by probing the heparin-bound eluents with an anti-PKR antibody specific for the kinase domain (**Supplementary Figure 3**).

**Figure 6:**
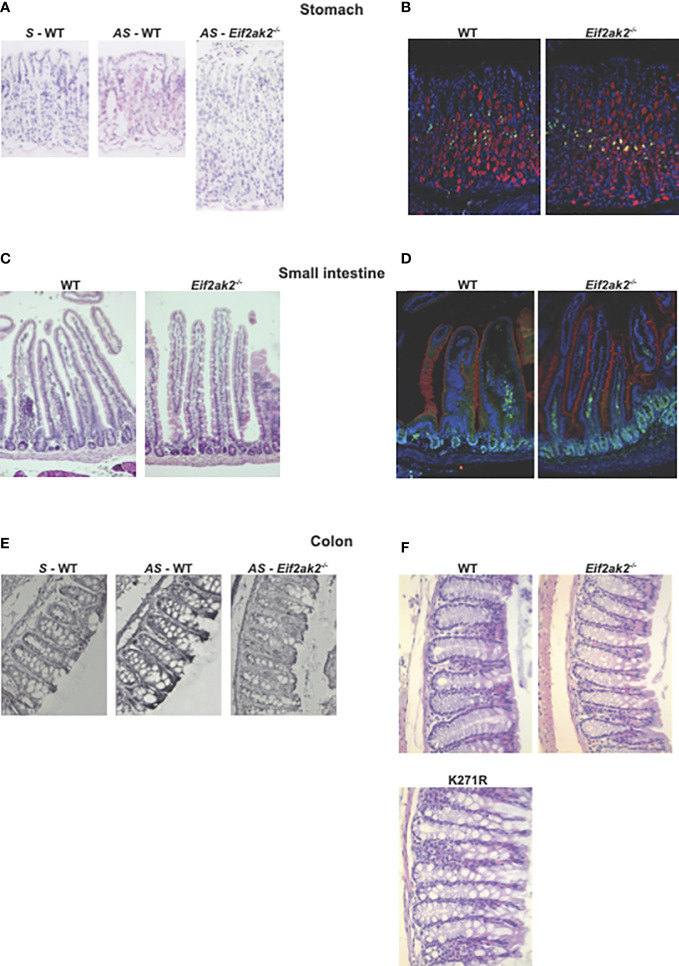
The effect of PKR on gastrointestinal physiology. **(A–F)** Micrographs of histologic specimens of the stomach, small intestine and colon from the indicated mice. **(A)** Tissue from the stomach probed with sense (S) and anti-sense (AS) oligonucleotides against the *Eif2ak2* transcript, and **(B)** stained for proliferating cell nuclear antigen (PCNA, green) and H^+^/K^–^ATPase (red) to assess cell proliferation and the parietal cells in the stomach mucosa, respectively, and counterstained with Hoechst (blue) to detect cell nuclei. **(C)** Tissue from the small intestine stained with H&E or **(D)** fluorescent probes against villin (red), to visualize microvilli at the brush boarder of the epithelial lining of the gut, PCNA (green) and Hoechst (blue). **(E)** Tissue from the colon probed with sense and anti-sense oligonucleotides against the *Eif2ak2* transcript and **(F)** stained with H&E to visualize the tissue structure. Representative images are shown from three independent experiments.

We then confirmed expression of the *Eif2ak2* transcript in the tissues from the stomach and colon by *in situ* hybridization (**Figures 6A, E**). Histological examination of tissue from throughout the gastrointestinal tract of the mice suggested a difference in goblet cells between the WT and PKR-ablated mice (**Figures 6B–F**). This appeared most evident in the small intestine (**Figure 6C**).

**Figure 7:**
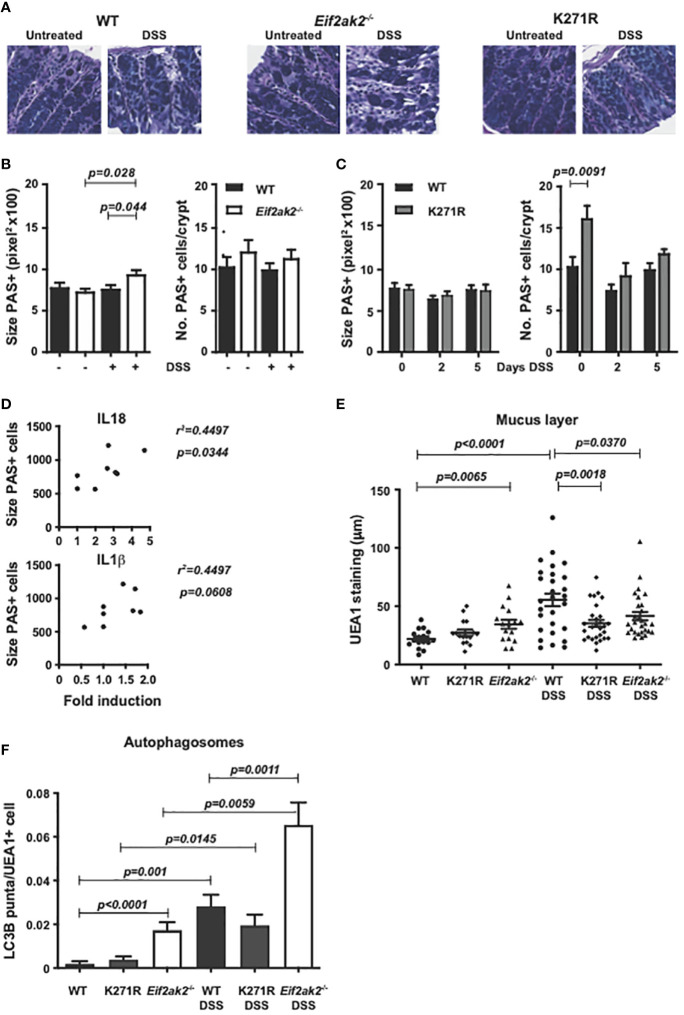
PKR alters gut physiology. **(A)** Micrographs of histologic specimens of the colon from WT, PKR-ablated (*Eif2ak2* ^-/-^) and kinase-dead (K271R) mice stained with PAS. Representative images are shown from two independent experiments. **(B, C)** Quantitation of the size and number of goblet cells visualized by PAS staining of colon from **(B)** WT and PKR-ablated (*Eif2ak2* ^-/-^) mice (H_2_O n=4 and DSS n=7) or **(C)** WT and kinase-dead (K271R) mice, either untreated or treated with DSS for 5 days or as indicated (H_2_O n=4 and DSS n=7). Data were analyzed by two-way ANOVA with Šidàk post-test. **(D)** Correlation analysis between the fold induction of IL18 or IL1β and the average goblet cell size from WT and PKR-ablated (*Eif2ak2* ^-/-^) mice (n=6). **(E)** Measures of the relative thickness of the mucus lining as visualized with UEA1 probing of colon tissues from the indicated mice, either untreated or treated with DSS in their drinking water for 5 days (H_2_O n=4 and DSS n=7). Each data point represents quantification of one field, with four to five fields assessed per confocal image per mouse. The data were analyzed by two-way ANOVA with Šidàk post-test. **(F)** Measures of autophagy were made by quantitation of LC3B puncta, identified as circular objects with a diameter of 10-70 pixels, in UEA1-positive cells from the indicated mice, either untreated or treated with DSS in their drinking water for 5 days (H_2_O n=4 and DSS n=7). 2000-4000 UEA1 positive cells were scored per microscopic field using CellProfiler software. Data are expressed as mean ± S.E.M. and analyzed by two-way ANOVA with Šidàk post-test.

Periodic acid-Schiff (PAS) staining of muco-substances supports a slight, non-statistically significant, increase in the number of goblet cells in the colon of PKR-ablated mice and a significant increase in the kinase-dead mouse compared to the WT animals (**Figures 7A–C**). PAS staining of colon tissue after 5 days of DSS treatment showed a significant increase in the size of goblet cells in the *Eif2ak2* ^-/-^ but not the kinase-dead or WT mice (**Figures 7A-C**). The goblet cell hyperplasia detected in the kinase-dead mouse resolved after DSS treatment (**Figure 7C**). Given our recognition that PKR controls inflammasome activity with previous reports linking goblet cell development with inflammasomes (19), we tested if there was a correlation between the size of goblet cells and the levels of the relevant cytokines. **Figure 7D** shows that the levels of IL18 (but not IL1β) correlated with goblet cell hypertrophy.

To further assess a consequence of changes to goblet cells, we assessed the mucus layer in the colon. *Ulex europaeus* agglutinin I fluorescein (UEA1) was used to visualize gastrointestinal fucosylated oligosaccharides. This stain detected that the mucus layer was reduced in the WT compared to the PKR-ablated and, to a lesser extent, the kinase-dead mice before exposure to DSS (**Figure 7E**). However, a substantive induction of mucin production in response to DSS treatment required PKR’s kinase activity (**Figure 7E**). Accordingly, the kinase activity of PKR appears to alter goblet cell physiology to promote stress-induced mucin production. This initial limitation but subsequent promotion in gut barrier function appears to correlate with the initial sensitivity but overall protection from DSS that was associated with PKR’s kinase activity.

As autophagy has been established to be essential for goblet cell function and because this response is regulated by eIF2α phosphorylation (20–22), we assessed this catabolic process in the different mice. Autophagy in goblet cells was assessed by probing the autophagic marker microtubule-associated protein 1B-light chain 3 (LC3B) in UEA1-positive cells. This measure detected more autophagic puncta in goblet cells from the PKR-ablated compared with either the WT or kinase-dead animals at homeostasis (**Figure 7F**). DSS treatment markedly induced the accumulation of autophagosomes in all the colon tissues while maintaining the differential between the separate genotypes (**Figure 7F**). It is important to recognize that this measure of autophagosome formation without a parallel assessment of lysozyme activity doesn’t capture autophagic flux, and so doesn’t detect an increase or decrease in the rate, but merely captures a change in autophagy (23).

Together these data identify that PKR alters the function of goblet cells in the gut, in part, by controlling autophagy. This activity is largely but not entirely dependent on substrate phosphorylation.

### PKR affects the gut flora

**Figure 8:**
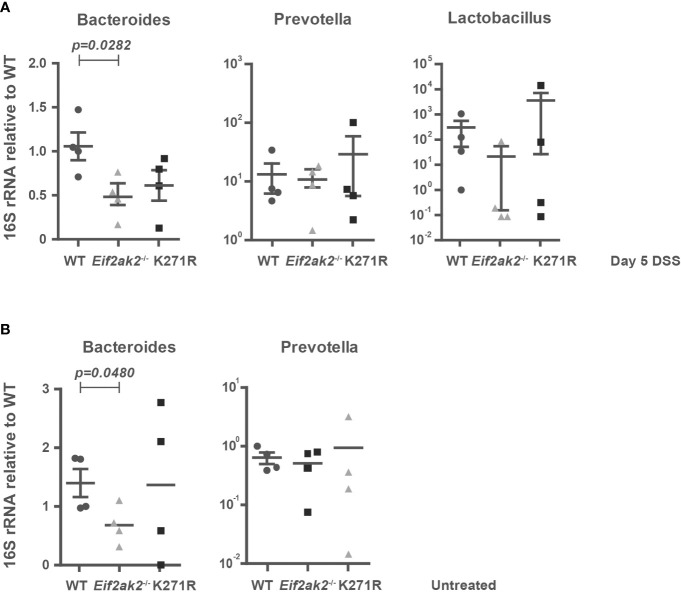
PKR affects the gut flora. **(A, B)** The amounts of *Bacteroides*, *Prevotella* and *Lactobacillus* species in fecal samples from WT, PKR-ablated (*Eif2ak2* ^-/-^) and kinase-dead (K271R) mice either **(A)** treated with DSS or **(B)** untreated (n=4). The quantities of bacteria were assessed by Q-PCR amplification of species-specific 16S rRNA and are expressed as fold induction of bacterial content compared to WT mice. The data are expressed as mean ± S.E.M. and analyzed by one-way ANOVA with Tukey’s range test.

As the intestinal mucus layer strongly influences the microbiome, we quantified specific microbes in the feces from WT, PKR-ablated and kinase-dead mice. Stool DNA was purified after 5 days of DSS treatment and used to amplify bacterial 16S rRNA sequences from the putatively colitogenic, gram-negative species *Bacteroides* and *Prevotella*, and the gram-positive species *Lactobacillus* by Q-PCR. Although the amounts of *Prevotella* and *Lactobacillus* species were equivalent among the three murine genotypes, *Bacteroides* species were significantly reduced in PKR mutant mice compared to the WT mice (**Figure 8A**). Examination of the stool from untreated mice shows that this is a pre-existing difference (**Figure 8B**). Accordingly, the levels of *Bacteroides*, which bind and metabolize mucins produced by goblet cells (24), correlate with PKR-dependent effects on mucin production.

## Discussion

We identify that PKR alters gut physiology to modify the response to DSS. Previous studies by Cao et al. and Rath et al. had showed that ablating PKR affects DSS-induced colitis but with discordant outcomes (6, 7). Our findings generally support those of Cao et al, which showed that PKR is protective against the weight loss from DSS treatment. However, in partial agreement with the findings of Rath et al, we detected an initial increase in DSS-induced tissue damage in mice with active PKR compared to mice that were ablated for the kinase. Consistent with the increased susceptibility, the mucin layer in the colon of mice expressing PKR was reduced at homeostasis. Nonetheless, a substantive induction of mucin in response to DSS required kinase activity and we identify that PKR altered autophagy in goblet cells in a kinase-dependent manner. Accordingly, PKR appeared to suppress barrier function at homeostasis but then promoted mucin production in response to challenge. These findings partly reconcile the previous discrepant findings and endorses a different mechanism of action for PKR in colitis than was asserted in the earlier studies by Cao et al. and Rath et al. Rather than PKR functioning by the UPR, we propose PKR functions by supporting gut barrier function *via* control of autophagy.

### A common function for different eIF2α kinases

Analogous to the PKR response shown here, investigations by Ravindran et al. showed that ablating another eIF2α kinase, GCN2, worsened weight loss in mice treated with DSS (8). This encourages the view that there may be a conserved response between different members of this kinase family. The response in GCN2-ablated mice was attributed to the control of inflammasome activity, with the DSS-induced weight loss able to be averted by also ablating inflammasome components or by antagonizing the related cytokine signaling. A similar activity is identified here for PKR and treating *Eif2ak2* ^-/-^ mice with an inhibitor of the NLRP3 inflammasome averted DSS-induced weight loss. This accords with a previous report identifying NLRP3 functions in DSS-induced colitis (25, 26). Neudecker et al. showed that, despite the benefit of the NLRP3 inhibitor, it did not reduce the level of IL18 in the colon of DSS-treated mice. This was replicated here, raising the question of how this inhibitor modifies the response. Our experiments implicated inflammasome activity in neutrophils in the response to DSS and numerous other studies support a contribution of neutrophils to colitis (4). However, the bone marrow chimera experiment conducted by Cao et al. identify that PKR function in epithelial cells is sufficient for the phenotype. Possibly in keeping with this we identify that PKR functions in goblet cells, which have been associated with NLRP3-dependent pathogenesis (27).

Inflammasome activity in GCN2-ablated mice was shown to stem from impaired autophagy in goblet cells (8). Autophagy is induced by eIF2α phosphorylation and Ravindran et al, with others, confirmed that mutation of the phosphoresidue of eIF2α exacerbated DSS-induced pathogenesis (20, 28). An earlier study had demonstrated that conditional expression of eIF2α with a mutated phosphoresidue in villus and crypt epithelial cells of the small and large intestine altered the susceptibility to DSS-induced colitis (29). This furthers the notion that the conserved activity of eIF2α kinases protect against DSS-induced colitis. However, our experiments with transgenic mice that express a kinase-dead PKR identify that the response to DSS is not entirely dependent on substrate phosphorylation. There may be some support for this in other studies, as the effect of mutating the phosphoresidue of eIF2α was less impactful than ablating GCN2 in the study by Ravindran et al. (8). Also, the protective effect of type III interferons in DSS-induced colitis was shown to be independent from the control of translation that is expected by eIF2α *phosphorylation* upon the induction of PKR expression (4). Significantly, ablating a third eIF2α kinase, PERK, did not affect the response to DSS treatment (8). As there is considerable evidence for the induction of the UPR in colitis, this appears to recognize that parallel responses, controlled by the activating transcription factor 6 or inositol-requiring enzyme 1α (IRE1α), can compensate for the loss of PERK independent of eIF2α phosphorylation (30, 31).

### An alternative mechanism of activity for PKR

The primacy of PERK in the eIF2α-mediated UPR, with the apparent ineffectiveness of ablating this kinase, somewhat weakens the proposal by Cao et al. and Rath et al. that PKR impacts DSS-induced colitis by control of the UPR. We propose an alternative mechanism of activity by control of autophagy. Our investigations detected an effect of PKR on goblet cell morphology and the production of mucin, as well as the levels of microbes that metabolize mucins. This equates with the mechanism of activity of GCN2 in DSS-induced colitis and is consistent with a previously identified function of eIF2α kinases in Paneth cells in the small intestine (29). Both cells are important for establishing barrier function, modulating the microbiota and the ensuing innate and acquired immunity (21, 24, 32). Notably, *Eif2ak2* ^-/-^ murine fibroblasts have defective autophagy and the expression of PKR rescued the starvation-induced autophagy in *GCN2*-disrupted yeast (33, 34). Accordingly, there is an established overlap in the responses controlled by these related kinases. However, our experiments suggest this can be separated to some extent from eIF2α phosphorylation.

Substrate phosphorylation-independent activities of PKR have been shown to be mediated through an association with the TNF receptor-associated factors (TRAFs) or the heat-shock protein (HSP) 70 and HSP90 (35–38). TRAFs shape signaling complexes and regulate the stability of the protein components by acting as adaptor molecules and ubiquitin ligases. Ablating TRAF proteins that interact with PKR affects autophagy and induces spontaneous colitis in mice (39–42). The HSP70 and HSP90 molecular chaperones also control autophagy and inflammasome activity, modulate DSS-induced colitis in mice, and have been shown to be protective in IBD (43–48). Possibly related to our speculation that other UPR proteins can offset the loss of PERK, IRE1α induces autophagy *via* TRAF2 and is also controlled by the HSP70 and HSP90 chaperones (49–51).

### Genetic difference as a cause of discrepant findings

The alternative mechanism that we propose may account for the discord between previous studies as the different murine strains used by Cao et al. (7) and Rath et al. (6) vary in their autophagic responses. The mice used by Cao et al. and ourselves were on an isogenic C57BL6/J background, while those used in the study by Rath et al. were on a mixed 129/terSv/BALB/C background. Autophagy is impaired in the BALB/C relative to the C57BL6/J strain (52–55). Among the consequences of this impairment is that DSS-induced damage to mitochondria would be predicted to accumulate through reduced mitophagy. This activates innate immune sensors, including PKR, that could cause the opposing activity that was reported (6, 55, 56). In addition to this defect in the BALB/C strain, the 129/terSv background is deleted for caspase-11 expression (57). Caspase-11 is protective in the context of DSS-induced colitis (58, 59), partly as a result of an autophagy-based secretory pathway for IL1β and IL18 (60–62). As the expression of caspase-11 is induced by PKR, through both eIF2α phosphorylation and kinase-independent signaling (14, 63–65), PKR expression would compound caspase-11-dependent differences in the responses of C57BL6/J and 129/terSv mice.

### Relevance for IBD

Autophagy is important in gastric function, particularly by supporting the function of secretory cells (66). Accordingly, PKR activity might be elicited to fortify gut barrier function and dampen immune pathogenesis in IBD. Type I and III interferons, which induce PKR expression and autophagy as well as suppressing inflammasome activity, are protective of DSS-induced colitis in mice (4, 5, 67, 68) and so might be trialled as a treatment for IBD. Notably, the more limited expression of the receptors for type III interferons mean that these cytokines are less prone to the contraindications of type I interferons (69, 70). The identification of kinase-substrate-independent activity in this study suggests therapeutic targets that would not induce the proteostasis that is induced by EIF2α phosphorylation. However, additional experiments are required to identify these targets. Inflammasome inhibitors have been suggested as potential therapies for immune pathogenesis. However, this is complicated by the positive functions of inflammasomes in gut immunity and wound healing that narrows the treatment window. Limiting inflammasome activity *via* autophagy may provide greater latitude with the broader benefits of cellular quality control. Towards this, autophagic inducers such as rapamycin and resveratrol have been shown to be beneficial in experimentally induced colitis. Other molecules that promote chaperone-mediated autophagy have shown promise in different diseases that share the pathogenic axis of deficient autophagy with elevated inflammasome activity that we propose as an etiology in IBD.

## Data availability statement

The original contributions presented in the study are included in the article/**Supplementary Material**. Further inquiries can be directed to the corresponding author.

## Ethics statement

The animal study was reviewed and approved by the Monash Medical Centre animal ethics committee and the Institutional Animal Care and Use Committee at the Cleveland Clinic.

## Author contributions

Conceptualization, AS. Methodology, HY, AC, SK, HM, DW, DS, AA and AS. Analysis and interpretation, HY, SK and AS. Manuscript preparation HY and AS. Funding acquisition, CM, RS, BW and AS. All authors contributed to the article and approved the submitted version.

## Supplementary material

Supplementary Figure 1 PKR-dependent pathogenesis in response to DSS. **(A, B)** Micrographs of histological specimens of colon tissue from WT and PKR-ablated (*Eif2ak2* ^-/-^) littermate mice from the Lerner Research Institute after 9 days of DSS treatment. Tissues are stained with α actin 2 (red), hyaluronic acid binding protein (green) and counterstained with DAPI (blue) in the upper panels and H&E in the lower panels. The PKR-ablated mice appear to display submucosae swelling compared to their WT littermates before treatment, suggesting a low level of basal inflammation (indicated by arrows). **(C)** Colitis scoring as a collated measure of submucosal swelling and angiogenesis below the expanded muscularis mucosae (visualized in red) and elevated hyaluronan deposition (visualized in green) in the upper panels and corruption of the rectal fold structure and loss of epithelial cells and crypts (visualized by H&E staining). **(D)** Calculation of the disease activity index (DAI) in mice treated with DSS.

Supplementary Figure 2 A comparison of proteins between the different PKR genotypes. Immunoblots comparing the relative expression of the indicated proteins between spleen cells from mice either untreated of treated with DSS (n=3) (*Eif2ak2* ^-/-^ and PKR-K271R symbolized as -/- and KR, respectively). Representative images from three independent experiments are shown.

Supplementary Figure 3 Confirmation of PKRs ablation in *Eif2ak2* ^-/-^ cells. An immunoblot with an anti-PKR antibody detecting PKR in whole-cell lysates and peptides captured with a heparin column from embryonic fibroblasts isolated from WT or the *Eif2ak2* ^-/-^ mice reported by Yang et al. (9) (on left) and the Coomassie-stained resolving gel after electrophoretic transfer to demonstrate the relative peptide levels (on right). The full-length PKR has been cleaved during this process so that the truncated kinase domain is the principal peptide detected in the WT lysates, while no PKR peptide is detected in the *Eif2ak2* ^-/-^ lysates.

## References

[1] Levin D London IM. Regulation of protein synthesis: Activation by double-stranded RNA of a protein kinase that phosphorylates eukaryotic initiation factor 2. Proc Natl Acad Sci U S A (1978) 75(3):1121–5. doi: 10.1073/pnas.75.3.1121 PMC411420 274704

[2] Jeon YJ Lim JH An S Jo A Han DH Won TB. Type III interferons are critical host factors that determine susceptibility to influenza a viral infection in allergic nasal mucosa. Clin Exp Allergy (2018) 48(3):253–65. doi: 10.1111/cea.13082 29288502

[3] Sommereyns C Paul S Staeheli P Michiels T. IFN-lambda (IFN-lambda) is expressed in a tissue-dependent fashion and primarily acts on epithelial cells in vivo. PloS Pathog (2008) 4(3):e1000017. doi: 10.1371/journal.ppat.1000017 18369468 PMC2265414

[4] Broggi A Tan Y Granucci F Zanoni I. IFN-lambda suppresses intestinal inflammation by non-translational regulation of neutrophil function. Nat Immunol (2017) 18(10):1084–93. doi: 10.1038/ni.3821 PMC5701513 28846084

[5] Katakura K Lee J Rachmilewitz D Li G Eckmann L Raz E. Toll-like receptor 9-induced type I IFN protects mice from experimental colitis. J Clin Invest (2005) 115(3):695–702. doi: 10.1172/JCI22996 15765149 PMC1051992

[6] Rath E Berger E Messlik A Nunes T Liu B Kim SC. Induction of dsRNA-activated protein kinase links mitochondrial unfolded protein response to the pathogenesis of intestinal inflammation. Gut (2012) 61(9):1269–78. doi: 10.1136/gutjnl-2011-300767 PMC4514769 21997551

[7] Cao SS Song B Kaufman RJ. PKR protects colonic epithelium against colitis through the unfolded protein response and prosurvival signaling. Inflammation Bowel Dis (2012) 18(9):1735–42. doi: 10.1002/ibd.22878 PMC3751177 22275310

[8] Ravindran R Loebbermann J Nakaya HI Khan N Ma H Gama L. The amino acid sensor GCN2 controls gut inflammation by inhibiting inflammasome activation. Nature (2016) 531(7595):523–7. doi: 10.1038/nature17186 PMC4854628 26982722

[9] Yang YL Reis LF Pavlovic J Aguzzi A Schafer R Kumar A. Deficient signaling in mice devoid of double-stranded RNA-dependent protein kinase. EMBO J (1995) 14(24):6095–106. doi: 10.1002/j.1460-2075.1995.tb00300.x PMC394734 8557029

[10] Yim HC Wang D Yu L White CL Faber PW Williams BR. The kinase activity of PKR represses inflammasome activity. Cell Res (2016) 26(3):367–79. doi: 10.1038/cr.2016.11 PMC4783464 26794869

[11] Kessler SP Obery DR de la Motte C. Hyaluronan synthase 3 null mice exhibit decreased intestinal inflammation and tissue damage in the DSS-induced colitis model. Int J Cell Biol (2015) 2015:745237. doi: 10.1155/2015/745237 26448758 PMC4581575

[12] Hornung V Bauernfeind F Halle A Samstad EO Kono H Rock KL. Silica crystals and aluminum salts activate the NALP3 inflammasome through phagosomal destabilization. Nat Immunol (2008) 9(8):847–56. doi: 10.1038/ni.1631 PMC2834784 18604214

[13] Okumura R Kurakawa T Nakano T Kayama H Kinoshita M Motooka D. Lypd8 promotes the segregation of flagellated microbiota and colonic epithelia. Nature (2016) 532(7597):117–21. doi: 10.1038/nature17406 27027293

[14] Bonnet MC Weil R Dam E Hovanessian AG Meurs EF. PKR stimulates NF-kappaB irrespective of its kinase function by interacting with the IkappaB kinase complex. Mol Cell Biol (2000) 20(13):4532–42. doi: 10.1128/MCB.20.13.4532-4542.2000 PMC85837 10848580

[15] Dagenais M Dupaul-Chicoine J Champagne C Skeldon A Morizot A Saleh M. A critical role for cellular inhibitor of protein 2 (cIAP2) in colitis-associated colorectal cancer and intestinal homeostasis mediated by the inflammasome and survival pathways. Mucosal Immunol (2016) 9(1):146–58. doi: 10.1038/mi.2015.46 26037070

[16] Coll RC Robertson AA Chae JJ Higgins SC Munoz-Planillo R Inserra MC. A small-molecule inhibitor of the NLRP3 inflammasome for the treatment of inflammatory diseases. Nat Med (2015) 21(3):248–55. doi: 10.1038/nm.3806 PMC4392179 25686105

[17] Baltzis D Li S Koromilas AE. Functional characterization of pkr gene products expressed in cells from mice with a targeted deletion of the n terminus or c terminus domain of PKR. J Biol Chem (2002) 277(41):38364–72. doi: 10.1074/jbc.M203564200 12161430

[18] Fasciano S Hutchins B Handy I Patel RC. Identification of the heparin-binding domains of the interferon-induced protein kinase, PKR. FEBS J (2005) 272(6):1425–39. doi: 10.1111/j.1742-4658.2005.04575.x PMC3969814 15752359

[19] Nowarski R Jackson R Gagliani N de Zoete MR Palm NW Bailis W. Epithelial IL-18 equilibrium controls barrier function in colitis. Cell (2015) 163(6):1444–56. doi: 10.1016/j.cell.2015.10.072 PMC4943028 26638073

[20] Humeau J Leduc M Cerrato G Loos F Kepp O Kroemer G. Phosphorylation of eukaryotic initiation factor-2alpha (eIF2alpha) in autophagy. Cell Death Dis (2020) 11(6):433. doi: 10.1038/s41419-020-2642-6 32513922 PMC7280501

[21] Patel KK Miyoshi H Beatty WL Head RD Malvin NP Cadwell K. Autophagy proteins control goblet cell function by potentiating reactive oxygen species production. EMBO J (2013) 32(24):3130–44. doi: 10.1038/emboj.2013.233 PMC3981139 24185898

[22] Lassen KG Kuballa P Conway KL Patel KK Becker CE Peloquin JM. Atg16L1 T300A variant decreases selective autophagy resulting in altered cytokine signaling and decreased antibacterial defense. Proc Natl Acad Sci U S A (2014) 111(21):7741–6. doi: 10.1073/pnas.1407001111 PMC4040621 24821797

[23] Tanida I Minematsu-Ikeguchi N Ueno T Kominami E. Lysosomal turnover, but not a cellular level, of endogenous LC3 is a marker for autophagy. Autophagy (2005) 1(2):84–91. doi: 10.4161/auto.1.2.1697 16874052

[24] Salyers AA Vercellotti JR West SE Wilkins TD. Fermentation of mucin and plant polysaccharides by strains of bacteroides from the human colon. Appl Environ Microbiol (1977) 33(2):319–22. doi: 10.1128/aem.33.2.319-322.1977 PMC170684 848954

[25] Neudecker V Haneklaus M Jensen O Khailova L Masterson JC Tye H. Myeloid-derived miR-223 regulates intestinal inflammation *via* repression of the NLRP3 inflammasome. J Exp Med (2017) 214(6):1737–52. doi: 10.1084/jem.20160462 PMC5460990 28487310

[26] Zaki MH Boyd KL Vogel P Kastan MB Lamkanfi M Kanneganti TD. The NLRP3 inflammasome protects against loss of epithelial integrity and mortality during experimental colitis. Immunity (2010) 32(3):379–91. doi: 10.1016/j.immuni.2010.03.003 PMC2982187 20303296

[27] McGilligan VE Gregory-Ksander MS Li D Moore JE Hodges RR Gilmore MS. Staphylococcus aureus activates the NLRP3 inflammasome in human and rat conjunctival goblet cells. PloS One (2013) 8(9):e74010. doi: 10.1371/journal.pone.0074010 24040145 PMC3769353

[28] B'Chir W Maurin AC Carraro V Averous J Jousse C Muranishi Y. The eIF2alpha/ATF4 pathway is essential for stress-induced autophagy gene expression. Nucleic Acids Res (2013) 41(16):7683–99. doi: 10.1093/nar/gkt563 PMC3763548 23804767

[29] Cao SS Wang M Harrington JC Chuang BM Eckmann L Kaufman RJ. Phosphorylation of eIF2alpha is dispensable for differentiation but required at a posttranscriptional level for paneth cell function and intestinal homeostasis in mice. Inflammation Bowel Dis (2014) 20(4):712–22. doi: 10.1097/MIB.0000000000000010 24577114

[30] Kaser A Lee AH Franke A Glickman JN Zeissig S Tilg H. XBP1 links ER stress to intestinal inflammation and confers genetic risk for human inflammatory bowel disease. Cell (2008) 134(5):743–56. doi: 10.1016/j.cell.2008.07.021 PMC2586148 18775308

[31] Brandl K Rutschmann S Li X Du X Xiao N Schnabl B. Enhanced sensitivity to DSS colitis caused by a hypomorphic Mbtps1 mutation disrupting the ATF6-driven unfolded protein response. Proc Natl Acad Sci U S A (2009) 106(9):3300–5. doi: 10.1073/pnas.0813036106 PMC2651297 19202076

[32] Roberton AM Stanley RA. *In vitro* utilization of mucin by bacteroides fragilis. Appl Environ Microbiol (1982) 43(2):325–30. doi: 10.1128/aem.43.2.325-330.1982 PMC241826 6174077

[33] Talloczy Z Jiang W Virgin H Leib DA Scheuner D Kaufman RJ. Regulation of starvation- and virus-induced autophagy by the eIF2alpha kinase signaling pathway. Proc Natl Acad Sci U S A (2002) 99(1):190–5. doi: 10.1073/pnas.012485299 PMC117537 11756670

[34] Shen S Niso-Santano M Adjemian S Takehara T Malik SA Minoux H. Cytoplasmic STAT3 represses autophagy by inhibiting PKR activity. Mol Cell (2012) 48(5):667–80. doi: 10.1016/j.molcel.2012.09.013 23084476

[35] Gil J Garcia MA Gomez-Puertas P Guerra S Rullas J Nakano H. TRAF family proteins link PKR with NF-kappa b activation. Mol Cell Biol (2004) 24(10):4502–12. doi: 10.1128/MCB.24.10.4502-4512.2004 PMC400457 15121867

[36] Horng T Barton GM Medzhitov R. TIRAP: an adapter molecule in the toll signaling pathway. Nat Immunol (2001) 2(9):835–41. doi: 10.1038/ni0901-835 11526399

[37] Donze O Abbas-Terki T Picard D. The Hsp90 chaperone complex is both a facilitator and a repressor of the dsRNA-dependent kinase PKR. EMBO J (2001) 20(14):3771–80. doi: 10.1093/emboj/20.14.3771 PMC125551 11447118

[38] Pang Q Christianson TA Keeble W Koretsky T Bagby GC. The anti-apoptotic function of Hsp70 in the interferon-inducible double-stranded RNA-dependent protein kinase-mediated death signaling pathway requires the fanconi anemia protein, FANCC. J Biol Chem (2002) 277(51):49638–43. doi: 10.1074/jbc.M209386200 12397061

[39] Piao JH Hasegawa M Heissig B Hattori K Takeda K Iwakura Y. Tumor necrosis factor receptor-associated factor (TRAF) 2 controls homeostasis of the colon to prevent spontaneous development of murine inflammatory bowel disease. J Biol Chem (2011) 286(20):17879–88. doi: 10.1074/jbc.M111.221853 PMC3093863 21393251

[40] Yang KC Ma X Liu H Murphy J Barger PM Mann DL. Tumor necrosis factor receptor-associated factor 2 mediates mitochondrial autophagy. Circ Heart Fail (2015) 8(1):175–87. doi: 10.1161/CIRCHEARTFAILURE.114.001635 PMC4303508 25339503

[41] Paul PK Kumar A. TRAF6 coordinates the activation of autophagy and ubiquitin-proteasome systems in atrophying skeletal muscle. Autophagy (2011) 7(5):555–6. doi: 10.4161/auto.7.5.15102 PMC3127217 21412053

[42] Shi CS Kehrl JH. TRAF6 and A20 regulate lysine 63-linked ubiquitination of beclin-1 to control TLR4-induced autophagy. Sci Signal (2010) 3(123):ra42. doi: 10.1126/scisignal.2000751 20501938 PMC6335036

[43] Tanaka K Namba T Arai Y Fujimoto M Adachi H Sobue G. Genetic evidence for a protective role for heat shock factor 1 and heat shock protein 70 against colitis. J Biol Chem (2007) 282(32):23240–52. doi: 10.1074/jbc.M704081200 17556362

[44] Collins CB Aherne CM Yeckes A Pound K Eltzschig HK Jedlicka P. Inhibition of n-terminal ATPase on HSP90 attenuates colitis through enhanced treg function. Mucosal Immunol (2013) 6(5):960–71. doi: 10.1038/mi.2012.134 PMC3748235 23321985

[45] Mayor A Martinon F De Smedt T Petrilli V Tschopp J. A crucial function of SGT1 and HSP90 in inflammasome activity links mammalian and plant innate immune responses. Nat Immunol (2007) 8(5):497–503. doi: 10.1038/ni1459 17435760

[46] Tanaka K Mizushima T. Protective role of HSF1 and HSP70 against gastrointestinal diseases. Int J Hyperthermia (2009) 25(8):668–76. doi: 10.3109/02656730903213366 20021227

[47] Martine P Chevriaux A Derangere V Apetoh L Garrido C Ghiringhelli F. HSP70 is a negative regulator of NLRP3 inflammasome activation. Cell Death Dis (2019) 10(4):256. doi: 10.1038/s41419-019-1491-7 30874540 PMC6420651

[48] Piippo N Korhonen E Hytti M Skottman H Kinnunen K Josifovska N. Hsp90 inhibition as a means to inhibit activation of the NLRP3 inflammasome. Sci Rep (2018) 8(1):6720. doi: 10.1038/s41598-018-25123-2 29712950 PMC5928092

[49] Urano F Wang X Bertolotti A Zhang Y Chung P Harding HP. Coupling of stress in the ER to activation of JNK protein kinases by transmembrane protein kinase IRE1. Science (2000) 287(5453):664–6. doi: 10.1126/science.287.5453.664 10650002

[50] Gupta S Deepti A Deegan S Lisbona F Hetz C Samali A. HSP72 protects cells from ER stress-induced apoptosis *via* enhancement of IRE1alpha-XBP1 signaling through a physical interaction. PloS Biol (2010) 8(7):e1000410. doi: 10.1371/journal.pbio.1000410 20625543 PMC2897763

[51] Marcu MG Doyle M Bertolotti A Ron D Hendershot L Neckers L. Heat shock protein 90 modulates the unfolded protein response by stabilizing IRE1alpha. Mol Cell Biol (2002) 22(24):8506–13. doi: 10.1128/MCB.22.24.8506-8513.2002 PMC139892 12446770

[52] Li CY Li C Li H Zhao GQ Lin J Wang Q. Disparate expression of autophagy in corneas of C57BL/6 mice and BALB/c mice after aspergillus fumigatus infection. Int J Ophthalmol (2019) 12(5):705–10. doi: 10.18240/ijo.2019.05.02 PMC6520259 31131226

[53] Breda J Banerjee A Jayachandran R Pieters J Zavolan M. A novel approach to single-cell analysis reveals intrinsic differences in immune marker expression in unstimulated BALB/c and C57BL/6 macrophages. FEBS Lett (2022) 596(20):2630–43. doi: 10.1002/1873-3468.14478 36001069

[54] Martyniszyn L Szulc-Dabrowska L Boratynska-Jasinska A Badowska-Kozakiewicz AM Niemialtowski MG. *In vivo* induction of autophagy in splenocytes of C57BL/6 and BALB/c mice infected with ectromelia orthopoxvirus. Pol J Vet Sci (2013) 16(1):25–32. doi: 10.2478/pjvs-2013-0004 23691572

[55] Pinheiro RO Nunes MP Pinheiro CS D'Avila H Bozza PT Takiya CM. Induction of autophagy correlates with increased parasite load of leishmania amazonensis in BALB/c but not C57BL/6 macrophages. Microbes Infect (2009) 11(2):181–90. doi: 10.1016/j.micinf.2008.11.006 19070676

[56] Mancini NL Goudie L Xu W Sabouny R Rajeev S Wang A. Perturbed mitochondrial dynamics is a novel feature of colitis that can be targeted to lessen disease. Cell Mol Gastroenterol Hepatol (2020) 10(2):287–307. doi: 10.1016/j.jcmgh.2020.04.004 32298841 PMC7327843

[57] Kayagaki N Warming S Lamkanfi M Vande Walle L Louie S Dong J. Non-canonical inflammasome activation targets caspase-11. Nature (2011) 479(7371):117–21. doi: 10.1038/nature10558 22002608

[58] Demon D Kuchmiy A Fossoul A Zhu Q Kanneganti TD Lamkanfi M. Caspase-11 is expressed in the colonic mucosa and protects against dextran sodium sulfate-induced colitis. Mucosal Immunol (2014) 7(6):1480–91. doi: 10.1038/mi.2014.36 PMC4205216 24850431

[59] Oficjalska K Raverdeau M Aviello G Wade SC Hickey A Sheehan KM. Protective role for caspase-11 during acute experimental murine colitis. J Immunol (2015) 194(3):1252–60. doi: 10.4049/jimmunol.1400501 PMC4298125 25548224

[60] Dupont N Jiang S Pilli M Ornatowski W Bhattacharya D Deretic V. Autophagy-based unconventional secretory pathway for extracellular delivery of IL-1beta. EMBO J (2011) 30(23):4701–11. doi: 10.1038/emboj.2011.398 PMC3243609 22068051

[61] Zhang M Kenny SJ Ge L Xu K Schekman R. Translocation of interleukin-1beta into a vesicle intermediate in autophagy-mediated secretion. Elife (2015) 4. doi: 10.7554/eLife.11205 PMC4728131 26523392

[62] Kimura T Jia J Kumar S Choi SW Gu Y Mudd M. Dedicated SNAREs and specialized TRIM cargo receptors mediate secretory autophagy. EMBO J (2017) 36(1):42–60. doi: 10.15252/embj.201695081 27932448 PMC5210154

[63] Endo M Mori M Akira S Gotoh T. C/EBP homologous protein (CHOP) is crucial for the induction of caspase-11 and the pathogenesis of lipopolysaccharide-induced inflammation. J Immunol (2006) 176(10):6245–53. doi: 10.4049/jimmunol.176.10.6245 16670335

[64] Kobori M Yang Z Gong D Heissmeyer V Zhu H Jung YK. Wedelolactone suppresses LPS-induced caspase-11 expression by directly inhibiting the IKK complex. Cell Death Differ (2004) 11(1):123–30. doi: 10.1038/sj.cdd.4401325 14526390

[65] Schauvliege R Vanrobaeys J Schotte P Beyaert R. Caspase-11 gene expression in response to lipopolysaccharide and interferon-gamma requires nuclear factor-kappa b and signal transducer and activator of transcription (STAT) 1. J Biol Chem (2002) 277(44):41624–30. doi: 10.1074/jbc.M207852200 12198138

[66] Bel S Hooper LV. Secretory autophagy of lysozyme in paneth cells. Autophagy (2018) 14(4):719–21. doi: 10.1080/15548627.2018.1430462 PMC5959324 29388875

[67] Schmeisser H Fey SB Horowitz J Fischer ER Balinsky CA Miyake K. Type I interferons induce autophagy in certain human cancer cell lines. Autophagy (2013) 9(5):683–96. doi: 10.4161/auto.23921 PMC3669179 23419269

[68] Guarda G Braun M Staehli F Tardivel A Mattmann C Forster I. Type I interferon inhibits interleukin-1 production and inflammasome activation. Immunity (2011) 34(2):213–23. doi: 10.1016/j.immuni.2011.02.006 21349431

[69] Davidson S McCabe TM Crotta S Gad HH Hessel EM Beinke S. IFNlambda is a potent anti-influenza therapeutic without the inflammatory side effects of IFNalpha treatment. EMBO Mol Med (2016) 8(9):1099–112. doi: 10.15252/emmm.201606413 PMC5009813 27520969

[70] Rauch I Hainzl E Rosebrock F Heider S Schwab C Berry D. Type I interferons have opposing effects during the emergence and recovery phases of colitis. Eur J Immunol (2014) 44(9):2749–60. doi: 10.1002/eji.201344401 24975266

