# Supplementary figures and images for: The protein kinase R modifies gut physiology to limit colitis
Source: Front Immunol. 2023 Feb 17;14:1106737. doi: 10.3389/fimmu.2023.1106737 (PMC9981792; doi:10.3389/fimmu.2023.1106737)

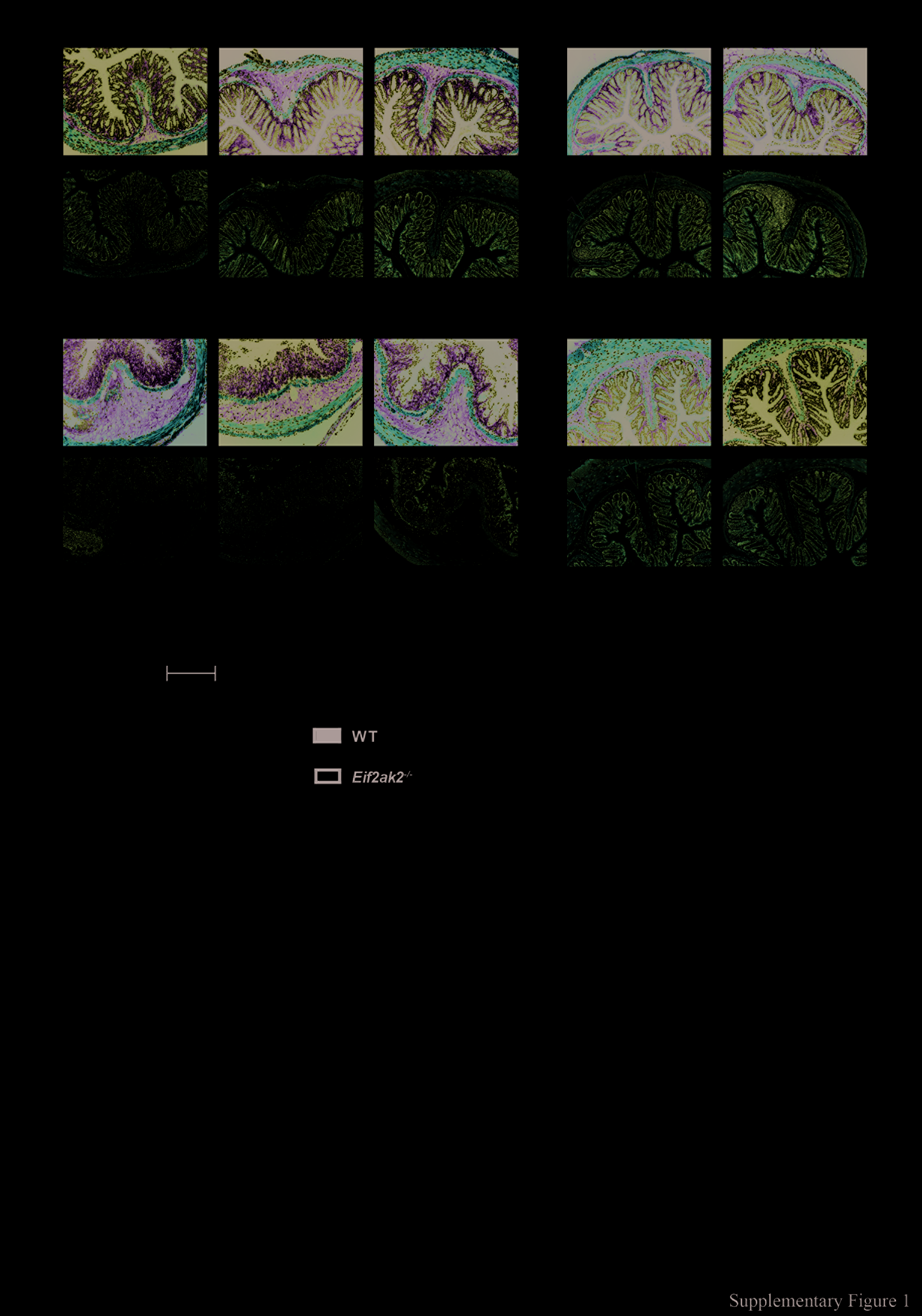

Supplement: Supplementary Figure 1 — PKR-dependent pathogenesis in response to DSS. (A, B) Micrographs of histological specimens of colon tissue from WT and PKR-ablated (Eif2ak2 -/-) littermate mice from the Lerner Research Institute after 9 days of DSS treatment. Tissues are stained with α actin 2 (red), hyaluronic acid binding protein (green) and counterstained with DAPI (blue) in the upper panels and H&E in the lower panels. The PKR-ablated mice appear to display submucosae swelling compared to their WT littermates before treatment, suggesting a low level of basal inflammation (indicated by arrows). (C) Colitis scoring as a collated measure of submucosal swelling and angiogenesis below the expanded muscularis mucosae (visualized in red) and elevated hyaluronan deposition (visualized in green) in the upper panels and corruption of the rectal fold structure and loss of epithelial cells and crypts (visualized by H&E staining). (D) Calculation of the disease activity index (DAI) in mice treated with DSS. [file Image_1.tif]

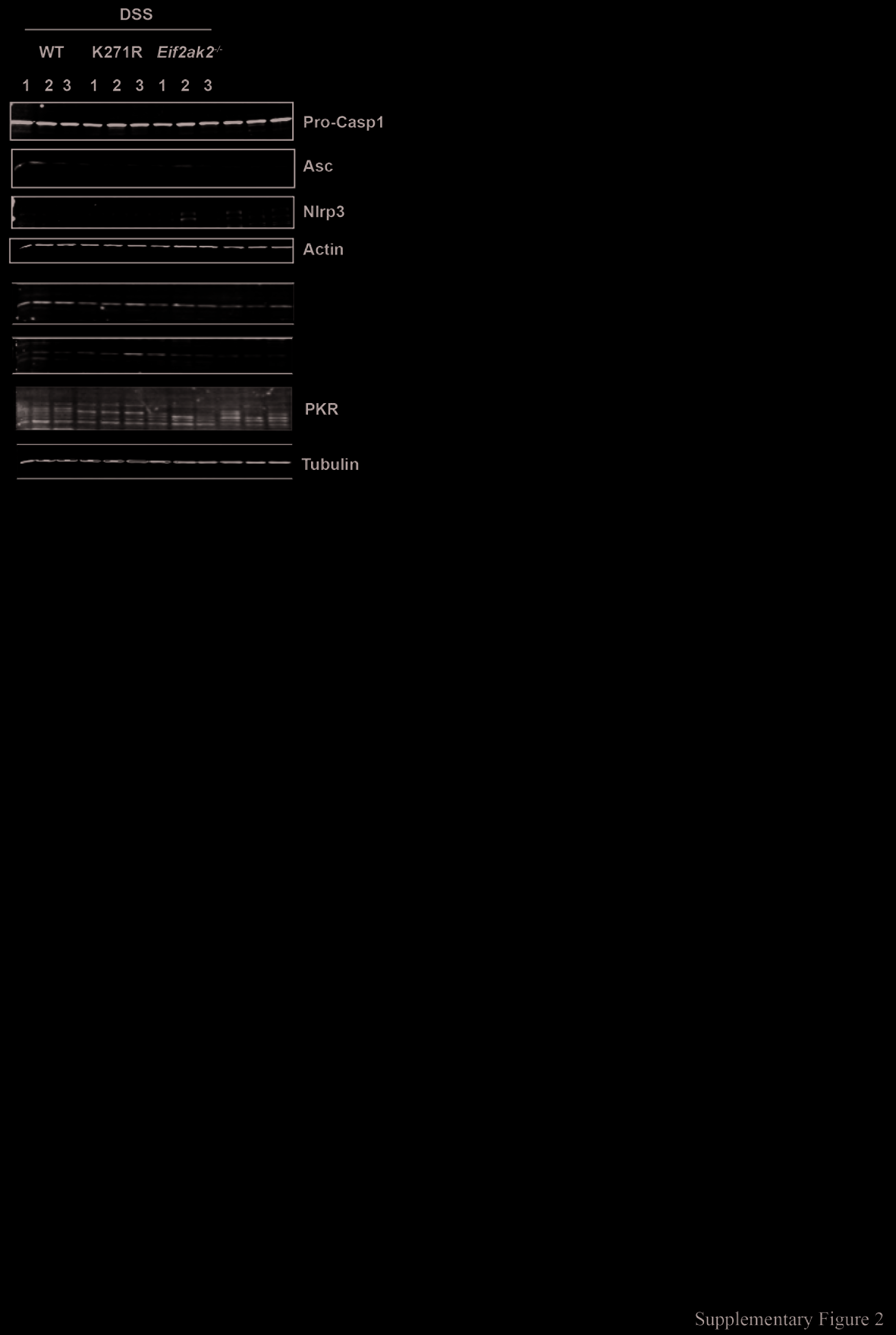

Supplement: Supplementary Figure 2 — A comparison of proteins between the different PKR genotypes. Immunoblots comparing the relative expression of the indicated proteins between spleen cells from mice either untreated of treated with DSS (n=3) (Eif2ak2 -/- and PKR-K271R symbolized as -/- and KR, respectively). Representative images from three independent experiments are shown. [file Image_2.tif]

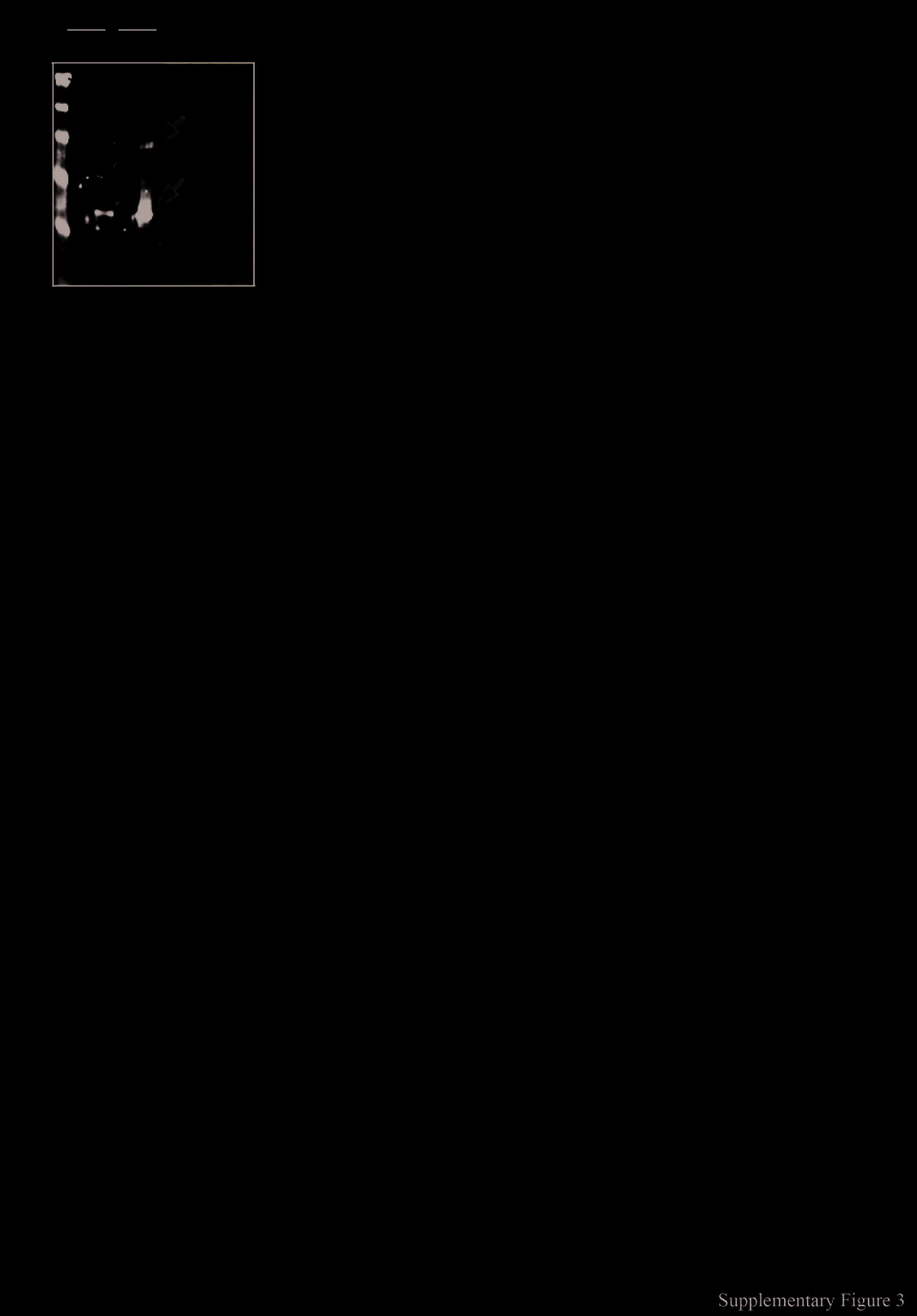

Supplement: Supplementary Figure 3 — Confirmation of PKRs ablation in Eif2ak2 -/- cells. An immunoblot with an anti-PKR antibody detecting PKR in whole-cell lysates and peptides captured with a heparin column from embryonic fibroblasts isolated from WT or the Eif2ak2 -/- mice reported by Yang et al. (9) (on left) and the Coomassie-stained resolving gel after electrophoretic transfer to demonstrate the relative peptide levels (on right). The full-length PKR has been cleaved during this process so that the truncated kinase domain is the principal peptide detected in the WT lysates, while no PKR peptide is detected in the Eif2ak2 -/- lysates. [file Image_3.tif]
